# Wireless Data Transmission at Terahertz Carrier Waves Generated from a Hybrid InP-Polymer Dual Tunable DBR Laser Photonic Integrated Circuit

**DOI:** 10.1038/s41598-018-21391-0

**Published:** 2018-02-14

**Authors:** Guillermo Carpintero, Shintaro Hisatake, David de Felipe, Robinson Guzman, Tadao Nagatsuma, Norbert Keil

**Affiliations:** 1 Universidad Carlos III de Madrid, Av. de la Universidad, 30. 28911 Leganés, Madrid, Spain; 2 Gifu University, Yanagido, Gifu 501-1193 Japan; 3 Fraunhofer Heinrich Hertz Institute, Photonics Components Department, Einsteinufer 37, 10587 Berlin, Germany; 4 Osaka University, Toyonaka, Osaka 560-8531 Japan

## Abstract

We report for the first time the successful wavelength stabilization of two hybrid integrated InP/Polymer DBR lasers through optical injection. The two InP/Polymer DBR lasers are integrated into a photonic integrated circuit, providing an ideal source for millimeter and Terahertz wave generation by optical heterodyne technique. These lasers offer the widest tuning range of the carrier wave demonstrated to date up into the Terahertz range, about 20 nm (2.5 THz) on a single photonic integrated circuit. We demonstrate the application of this source to generate a carrier wave at 330 GHz to establish a wireless data transmission link at a data rate up to 18 Gbit/s. Using a coherent detection scheme we increase the sensitivity by more than 10 dB over direct detection.

## Introduction

The 5 G revolution is the common form to refer to the new generation of network technologies, which are expected to have a profound transformation of society and industry. Although 5 G is not yet fully standardized, some of the key specifications have already been established, requiring data connections well above 10 Gbit/s with latency below 5 milliseconds^1^. The high data rates involved require the deployment of high density small cells, especially in urban environment. Providing backhaul connectivity to the small cells with the required capillary and data rate is a challenge, attracting the use of carrier waves in the millimeter-wave (MMW, 30 to 300 GHz) and Terahertz wave (THz, 300 GHz to 3 THz) frequency range to support multigigabit data rate. These needs are driving regulatory bodies to reconsider the spectrum allocation. As an example, just recently Japan allocated the range from 116 GHz to 134 GHz (18 GHz of bandwidth) for point to point wireless links^2^. Further above, the range from 275 GHz to 300 GHz has not yet been allocated, where 25 GHz of bandwidth are available^3^.

To date, most wireless data transmission demonstrations reporting data rates beyond 10 Gbit/s employed carrier wave frequencies above 100 GHz generated with photonic techniques^3^. Photonic-based technologies are leading the access to the high frequency range, offering several key advantages such as use optical fibers for low-loss distribution of high-frequency RF signals over long distances, provide Electro-Magnetic Interference (EMI) immunity and allow the seamless integration of fiber and wireless networks^4^. Another key advantage is the availability of telecom-based high-frequency components, such as lasers, modulators and opto-electronic (O-E) converters (photodiodes) with large modulation bandwidths. However, as the requirements increase for data rate and link distance, coherent wireless communication systems must be used. These require extremely stable carrier waves^5^, which become an issue when semiconductor lasers are used in their generation.

Currently there is a plethora of photonic-based MMW and THz signal generation techniques, each having different performance in terms of maximum achievable frequency, frequency tuning range and frequency stability. However, it is a challenge to achieve a signal source capable of generating a carrier wave frequency within the full MMW and THz range, tunable over the entire range, without sacrificing its spectral purity. Optical heterodyning is the technique that offers the simplest mechanism with the widest frequency tuning range. This technique relies on generating two optical wavelengths, which beat into a photodiode to generate an electrical signal with a frequency determined by the wavelength spacing. However, to achieve a high purity signal, the phase noise of these two wavelengths must be mutually correlated.

One option is to use an optical frequency comb generator (OFCG). If the OFCG output signal illuminates a high-speed photodiode, we generate an electrical frequency comb with lines located at the fundamental frequency given by the wavelength line spacing as well as its harmonics. When a single, spectrally pure electrical signal is desired, this requires that only two wavelengths be derived from the OFCG output. This is the approach recently used to generate a carrier wave at 237.5 GHz for a wireless communication system, transmitting at 100 Gbit/s, the highest reported to date^6^. The OFCG was a solid-state mode locked laser, with a fixed wavelength spacing of 12.5 GHz. However, to select two wavelengths from the comb, the OFCG needs to be followed by a bulky and costly programmable tunable optical filter, which does not easily turn into a compact system. Passive filters, such as the array-waveguide grating and the fiber Bragg grating have been developed, but neither their tuning range is wide nor their response fast^7^.

An alternative is to use two single frequency tunable laser diodes subject to a phase locking technique, either through phase locked loops^8^ or optical injection^9^. Since phase locked loops are limited in the tuning range by the loop delay to frequencies below 50 GHz, optical injection locking (OIL) of a laser diode is a preferred technique^9^. OIL is a promising technique toward the widely tunable and fast optical filter. In addition, the single frequency tunable laser diode, as an active device, does not just select the wavelength but it also amplifies the optical power level.

An approach that is revolutionizing photonic-based millimeter and Terahertz wave wireless communications is the use of photonic integration techniques, developing the photonic-based signal source on a single chip, as a Photonic Integrated Circuit (PIC). Integrating the key components into a single chip provides key advantages of reducing the optical path lengths among the components as well as enabling all of them and their connecting optical waveguides to experience the same temperature fluctuations, which has also demonstrated to be key for the reduction of the phase noise^10^. To date, compact and light millimeter-wave transmitter front-ends have been demonstrated based on integrated optical heterodyne signal generation using different integration technology platforms, monolithic InP PICs^11^ and heterogeneous silicon-InP^12^, integrating two tunable lasers, showing a tuning range about 100 GHz. To the best of our knowledge we present for the first time a Terahertz wireless link in which a 330 GHz carrier wave is generated using an optical heterodyne signal source based on two tunable hybrid InP/Polymer distributed Bragg reflector (DBR) lasers from a hybrid polymer integration platform. We also demonstrate for first-time wavelength stabilization of the tunable hybrid InP/Polymer distributed Bragg reflector (DBR) lasers through optical injection and their use within a coherent wireless transmission link.

## Results

### Device Description

**Figure 1:**
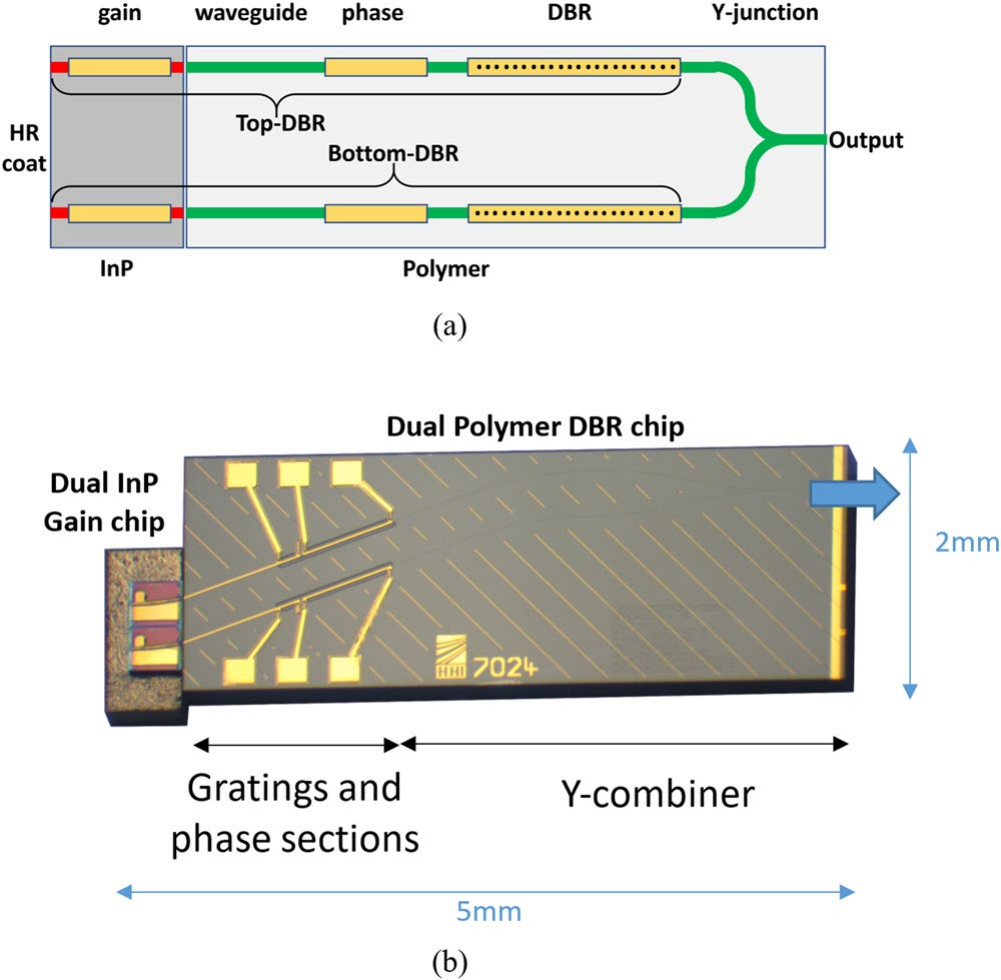
Hybrid integrated dual wavelength source chip, (**a**) Schematic showing the gain sections on InP and the elements on the polymer substrate, including passive waveguides, phase shifters and DBR sections and (**b**) photograph of the dual hybrid InP/Polymer DBR.

The tunable hybrid InP/Polymer DBR is a novel kind of cost-effective and yet high-performance tunable laser source available at the PolyBoard platform. The major advantage of using this platform is that it is highly flexible to combine the advantages of various material platforms. Details about the fabrication of the lasers and the wide range of components that can be integrated on the platform can be found at^13^. With regard to the used hybrid InP/Polymer DBR lasers, a schematic of the structure is presented in Fig. 1(a), showing a dual wavelength source integrating two tunable hybrid InP/Polymer DBR lasers using an InP-based active chip and a Polymer-based passive chip.

The InP-based gain chip acts as optical gain medium and includes two 400 μm long gain sections, one for each of the lasers. Each gain section is a buried-heterostructure (BH)-type gain chip, with MOVPE grown InGaAsP multi-quantum well active region and pn-InP blocking layers. The photoluminescence wavelength is 1570 nm. The cleaved facets of the chip have a high-reflection (HR) coated left-hand facet and an anti-reflection (AR) coated right-hand facet. Further, the gain sections are angled 9° with respect to the right-hand facet to avoid undesired reflections at the InP-polymer interface. To obtain high mode overlap between the InP gain chip and the polymer waveguide (i.e. low coupling loss), the gain chip waveguide is laterally tapered down to about 0.4 μm at the right-hand facet^14^.

The Polymer chip uses passive buried channel waveguides with square cross section fabricated with the polymer material ZPU-12 (perfluorinated oligomer and acrylate) from ChemOptics Inc. This material is spin-coated on a standard 4-inch or 6-inch silicon wafer, cured under UV illumination, and baked at 200 °C during 30 min. Waveguides are structured with conventional photolithography followed by reactive ion etching (RIE)^15^. On the polymer, the hybrid InP/Polymer DBR lasers integrate phase and grating sections. The phase section, 200 μm long in our device, allows for precise tuning of the laser wavelength and control the side-mode suppression ratio (SMSR). The grating section has a 1000 μm long distributed Bragg reflector, using 3rd-order sidewall-corrugated Bragg grating, for wavelength selective feedback mirror. In addition, the polymer chip includes a Y-junction to combine the wavelengths of the two lasers.

Figure 1(b) shows a picture of the hybrid integrated chip, where the InP and Polymer chips are butt-coupled. The laser resonator is defined between the DBR section (with the wavelength selective mirror on the right) and the high-reflection (HR) coating of the left-hand facet of the InP gain chip. These lasers have an extremely wide frequency tuning (wavelength shifting) range in excess of 20 nm (~2.5 THz)^13^.

**Figure 2:**
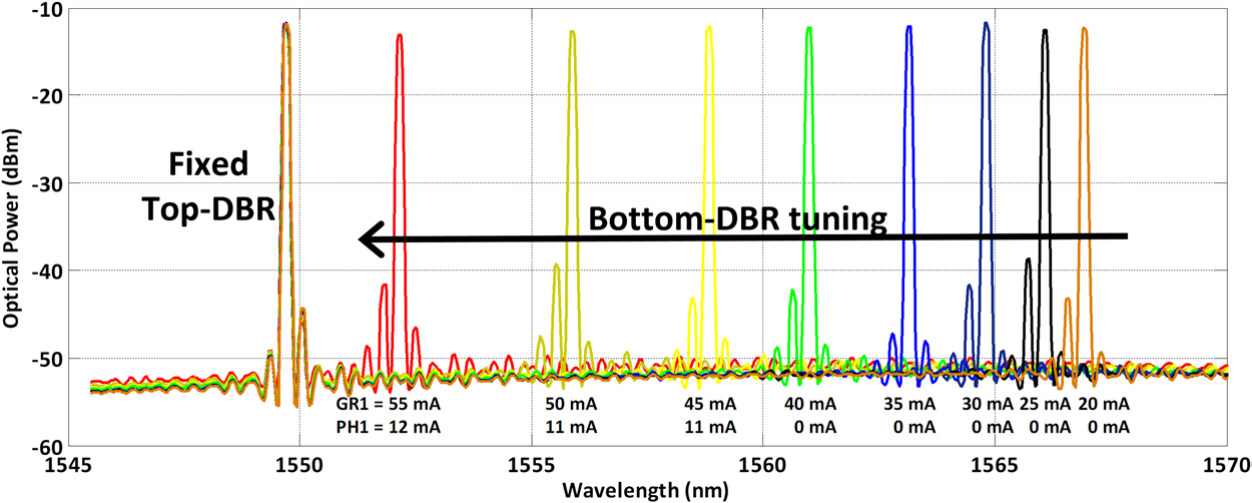
Wavelength tuning of the InP polymer DBR laser, demonstrating a wide tuning range from 18.55 nm (2.25 THz) to 2.64 nm (330 GHz), varying the current applied to the grating section (GR) and the phase section (PS).

The Bragg period of each InP/polymer DBR grating is designed so that the two laser wavelengths start lasing at the wavelengths shown in Fig. 2, 1550 nm and 1568 nm. The wavelengths of each laser can be thermos-optically tuned through heater electrodes in the DBR and phase sections, as well as through the current injected in the gain section. As shown in this Fig. 2, varying the current on the grating section (GR) from 20 mA to 55 mA the wavelength varies from 1568 nm to 1552.64 nm, establishing a spacing between the two lasers of 2.64 nm (330 GHz). This is the carrier frequency for our coherent wireless transmission link that has been considered in this work.

### Terahertz signal generation

**Figure 3:**
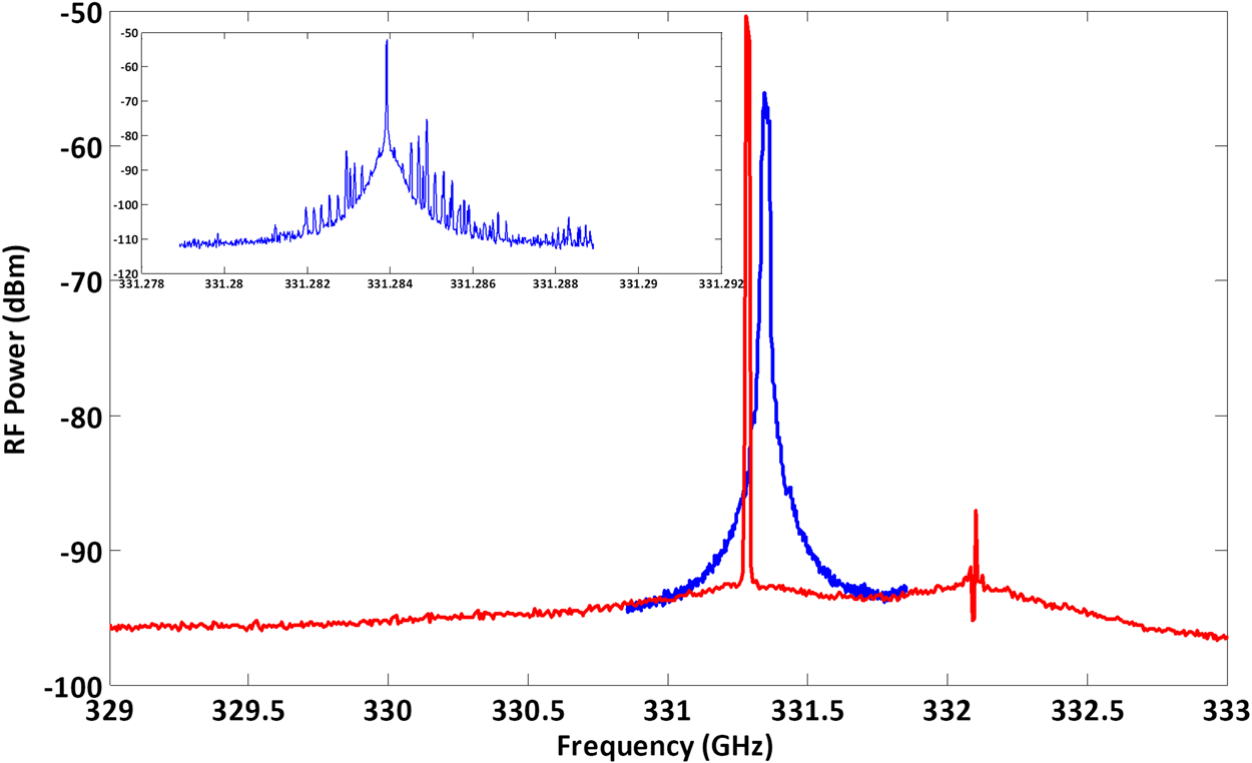
Electrical spectra of the photonic generated beat note at 330 GHz. The linewidth is reduced under the injection locking condition. The slight frequency difference between the free-running (blue) and the injection-locked (red) is caused by the optical power injected into the DBR lasers. RBW 2 MHz, VBW 3 kHz. The inset shows the injection-locked beat note with RBW 6 kHz.

The first step is to set the bias conditions in the two hybrid InP/Polymer DBR lasers to fix the wavelength spacing to 330 GHz and measure the generated beat note signal in both, a free-running condition and with optical injection from the external OFCG. The beat note was produced by a uni-traveling-carrier photodiode module (UTC-PD IOD-PMJ-13001, NTT Electronics) with an operating frequency range from 280 to 380 GHz and center frequency of 330 GHz (limited by the rectangular waveguide output) followed by a sub-harmonic mixer (Virginia Diodes Inc.) to down-converted the signal into the range of the electrical spectrum analyzer (Rohde & Schwartz FSV40-B21). As shown in Fig. 3, the electrical beat note in free-running conditions (blue trace) shows a full-width half-maximum (FWHM) linewidth of 2.8 MHz. This value is comparable to the best case for monolithically integrated dual DFB lasers^11^, and is sufficient for wireless communication links using direct detection as well as for spectroscopy.

The OFCG used for optical injection locking is based on a single frequency tunable seed laser (TL), emitting 0 dBm optical power at 1551.05 nm. The TL output is followed by two cascaded Electro-Optic Phase Modulators (EOPM), fed from a Rohde & Schwartz Continuous Wave (CW) with modulation frequency set to *f*_*m*_ = 27.5 GHz, providing 12.7 dBm of RF power. The frequency is selected as an integer sub-multiple of the hybrid InP/Polymer DBR lasers wavelength difference (27.5 GHz × 12 = 330 GHz). The OFCG output is fed to the InP/Polymer DBR lasers through a Variable Optical Attenuator (VOA) to select the amount of optical injection power, followed by an in-line Optical Power Monitor (OPM) to measure it, and an Optical Circulator (OC) to separate the injected signal from the dual wavelength output of the dual InP/Polymer DBR PIC.

Applying −8.97 dBm of optical injection power into the dual InP/Polymer DBR PIC, the generated signal linewidth reduces to about 12 kHz (red trace in Fig. 3), limited by the instrument minimum resolution bandwidth. The linewidth reduction is due to the fact that the InP/Polymer DBR lasers integrated in the PIC inherit the stability of the CW source used to create the comb, and the beat note is fixed at the OFCG line spacing, 330 GHz.

### Wireless Data Link Demonstration

**Figure 4:**
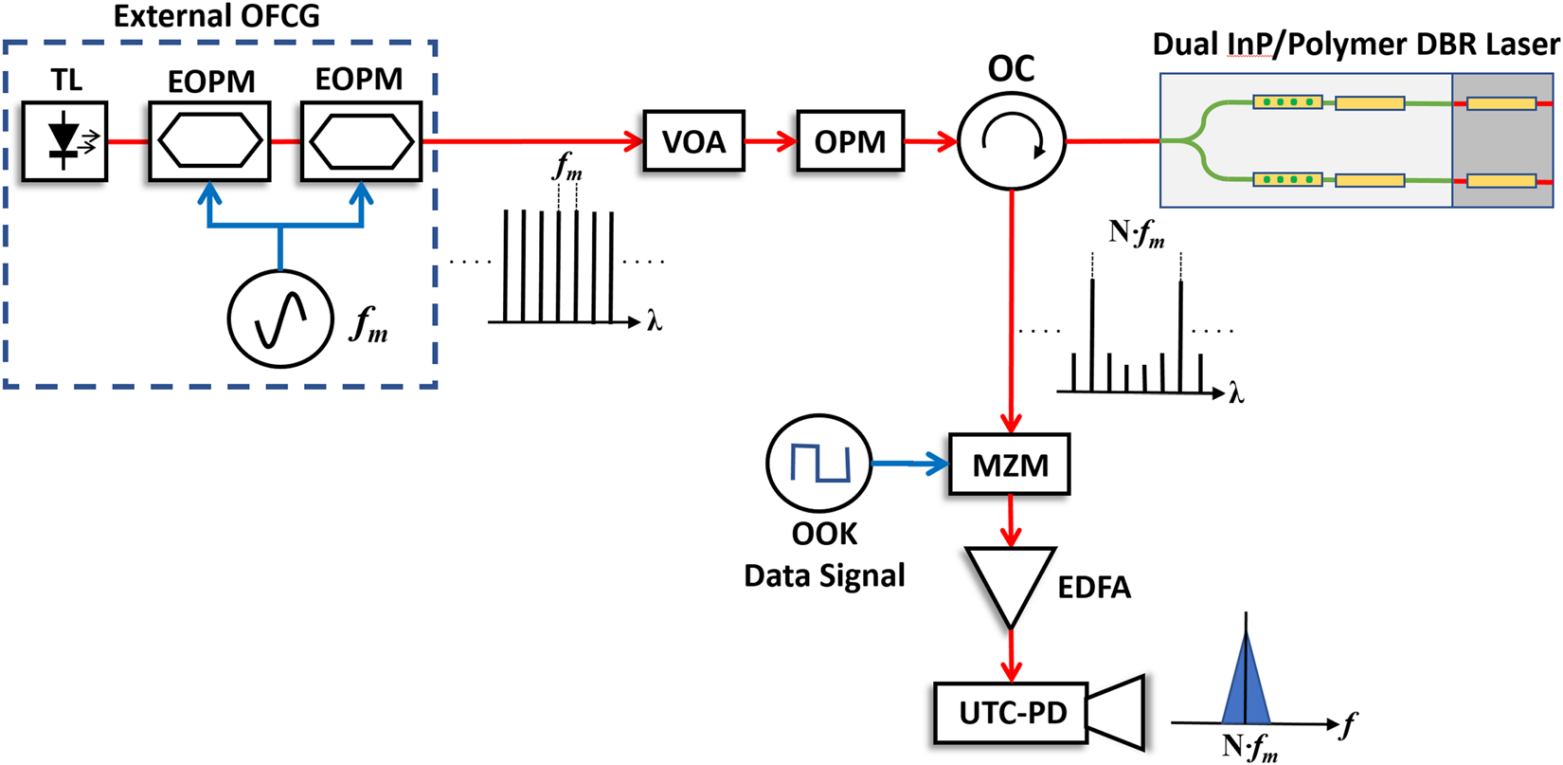
Schematic of the experimental setup for optical injection locking of the hybrid dual InP/Polymer DBR Photonic Integrated Circuit for coherent photonic wireless transmitter. OFCG: Optical Frequency Comb Generator, EOPM: Electro-Optic Phase Modulator, MZM: Mach-Zehnder Modulator, EDFA: Erbium-Doped Fiber Amplifier, UTC-PD: Uni-Traveling Carrier Photodiode, VOA: variable optical attenuator, OPM: optical power monitor.

To fully demonstrate the potential of the stabilized source, we developed a wireless communication link with coherent detection. On the transmitter, shown in Fig. 4, we used a Mach-Zehnder optical intensity modulator. The modulator is located at the output of the InP/Polymer chip, and therefore the two independent optical carriers (separated at the desired mm-wave frequency) are simultaneously modulated. It has been shown that this is equally effective to the scheme where instead of the two optical carriers only one is modulated^16^.

**Figure 5:**
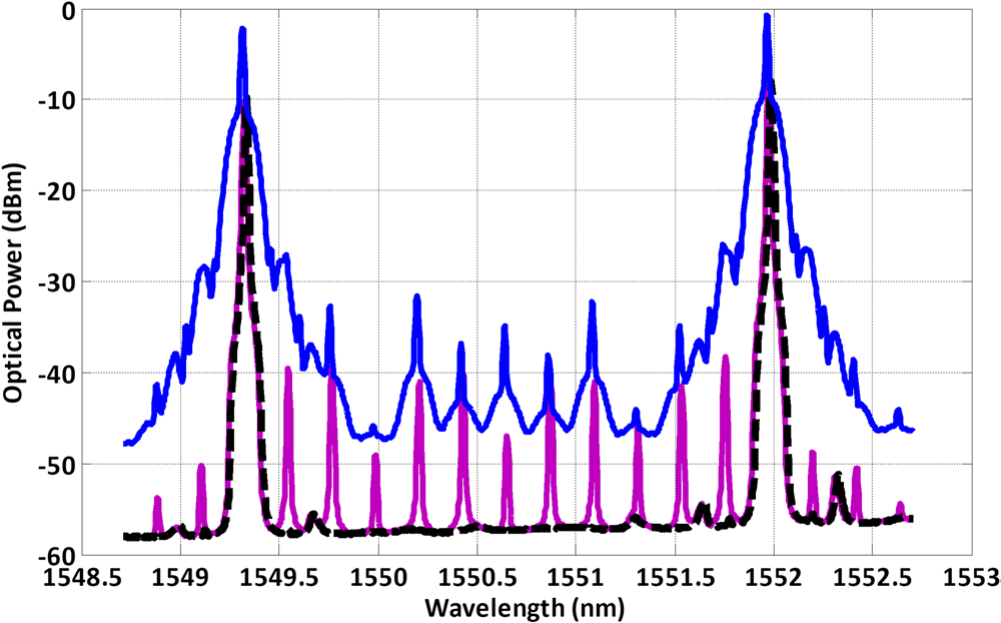
Optical spectrum of the hybrid InP/Polymer dual DBR Photonic Integrated Circuit with 330 GHz spacing between wavelengths under different conditions: (dashed black) free-running, (pink) with optical injection from the external OFCG, and (blue) after the MZM, with a pseudo-random bit data stream at 18 Gbit/s.

The data signal is an ASK modulation format from a pattern generator system. The data stream was a pseudo random bit sequence with a length of 2^15^ − 1 at a bit rate of 18 Gbit/s. An erbium-doped fiber amplifier (EDFA) and an UTC-PD converted the optical signal into a 330 GHz modulated carrier wave. The generated RF signal is radiated from the horn antenna. Figure 5 shows the optical spectrum from the hybrid InP/Polymer dual DBR PIC, measured with an AQ6370C Optical Spectrum Analyzer (OSA). As the maximum resolution is 20 pm (2.5 GHz), and the optical linewidth can be estimated to be half of the electrical beat note in free-running conditions (2.8 MHz), all the optical lines seem to have the same linewidth on the OSA. The dashed black trace on Fig. 5 shows the two optical wavelengths, each corresponding to a InP/Polymer DBR laser on the PIC under free-running condition. The spacing between the two wavelengths has been adjusted to 330 GHz through the gain (20 mA), phase (12 mA) and grating (55 mA) section currents. Finally, the pink trace of Fig. 5 shows the optical spectrum when the InP/Polymer dual DBR PIC with an optical injection power of −8 dBm. The comb lines between the two InP/Polymer dual DBR wavelengths have a side mode suppression ratio higher than 30 dB. After the MZM, the optical spectrum shows that every wavelength in the spectrum is modulated.

The receiver, separated from the transmitter 45 mm for easy alignment, used a sub-harmonic mixer (SHM). The intermediate frequency (IF) signal was amplified by a pre-amplifier (MITEQ JSMF4; 3 kHz-18 GHz) and reshaped by a trans-impedance amplifier (Gigoptix). The BER characteristics were measured in real-time using a bit error rate tester (BERT, Anritsu: MP1776A) and the eye diagrams with an oscilloscope (Agilent: 86100 A).

**Figure 6:**
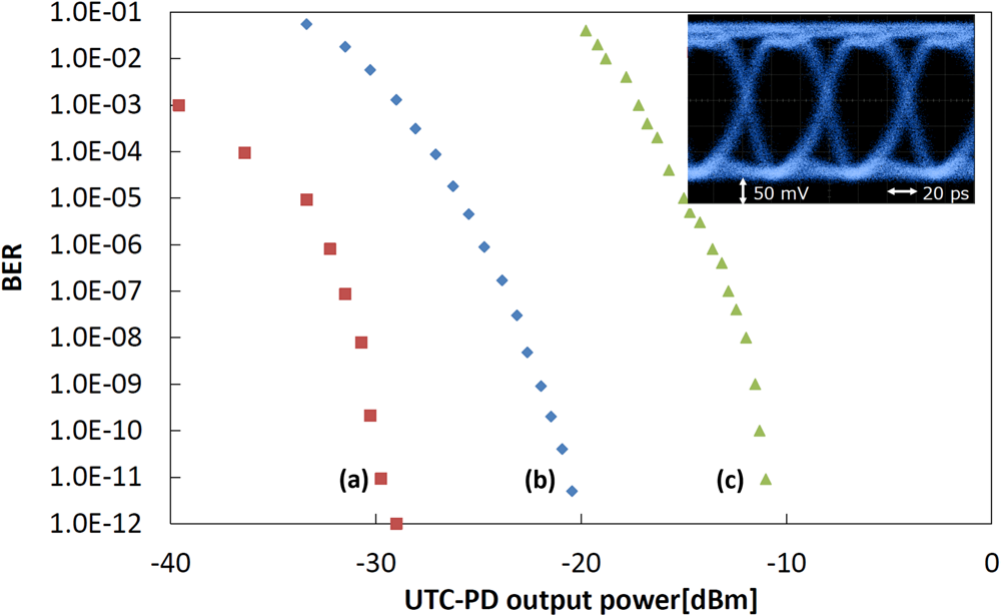
Bit error rate (BER) characteristics at 18 Gbit/s generating the 330 GHz carrier from: (**a**) an actively phase stabilized reference source^18^, (**b**) the dual hybrid InP/Polymer DBR with −8 dBm optical injection power from the OFCG, and (**c**) free-running dual hybrid InP/Polymer DBR lasers and using direct detection at receiver. The inset shows the eye diagram under conditions of (**b**).

In a first approach, for the sake of comparison, the wireless link has been operated without frequency stabilization. With the lasers in a free-running condition, we used a direct detection scheme using a Schottky-barrier diode detector. Using this scheme, as shown in Fig. 6(c), we achieved a 10^−11^ BER for a transmitted output power of −10 dBm from the UTC-PD.

The next step was to use the optical injection locking stabilization of the pair of DBR lasers in order to introduce a coherent receiver scheme. In this proof-of-concept experiment, the LO signal at the receiver mixer was supplied from the same synthesizer used for the OFC generator (OFCG). This is to reduce the impact of the phase noise of the synthesizer on the system so that we can evaluate our coherent transmitter in an ideal condition. In this case, we achieved a BER of 10^−11^ for a transmitted output power of −20 dBm from the UTC-PD. Compared to the free-running lasers and direct detection, this provides a 10 dB sensitivity improvement. It is worth mentioning that error-free transmission was achieved even though the OFC spacing was 27.5 GHz and the data rate 18 Gbit/s.

We must highlight the fact that the OFCG output is directly fed into the InP/Polymer DBR lasers, without any optical filter to select the comb lines prior to the optical injection. As shown by the blue trace of Fig. 5, we achieve a Side Mode Suppression Ratio (SMSR) of 30 dB between the InP/Polymer DBR laser lines and the other OFCG lines. Since all these wavelengths pass through the data modulator, the modulation bandwidth enlarges the spectrum of each one of them. As the data rate increases, the modulation sidebands among adjacent lines overlap. We believe this overlap is responsible for the 9 dB power penalty in the BER characteristics incurred by the dual hybrid InP/Polymer DBR source compared to the actively phase stabilized reference source^17^.

## Discussion

In summary, we have demonstrated the stabilization of the two wavelengths generated from a novel dual wavelength source based on hybrid integration of two InP/Polymer DBR lasers through optical injection locking. These lasers offer an extremely wide wavelength tuning range, potentially enabling to generate any frequency within the microwave (3 GHz to 30 GHz), millimeter-wave (30 GHz to 300 GHz) and into the Terahertz (300 GHz to 3000 GHz) ranges. Using this stabilized dual wavelength source, the linewidth of the beat note reduces from tens of MHz to tens of kHz, which has enabled to demonstrat error free data transmission in a wireless communication link operating at 330 GHz, showing a BER of 10^−11^ with a transmitter power of −20 dBm generated at the transmitter UTC-PD. The fact that the hybrid integration platform offers a wide range of components that can be integrated together (e.g. the optical circulator), turn it into an extremely promising platform to develop the components for real-time ultrafast coherent wireless transmitters.
